# Knowledge, attitudes, and training on genetic testing among public health professionals and students in the United Arab Emirates: a qualitative study

**DOI:** 10.1186/s12909-026-09262-z

**Published:** 2026-04-29

**Authors:** Yasir Ahmed Mohammed Elhadi, Ismail Elkonaisi, Meera Alneyadi, Bahar Kasaei, Bayan Hassan, Maryam Alkaabi, Aysha Althehli, Iffat Elbarazi, Fatma Al-Maskari, Azhar T. Rahma

**Affiliations:** 1 Institute of Public Health, College of Medicine & Health Sciences, United Arab Emirates University, Al-Ain, 15551 United Arab Emirates; 2 Birmingham University Dubai, Dubai, United Arab Emirates; 3 Zayed Center for Health Sciences, United Arab Emirates University, Al-Ain, United Arab Emirates

**Keywords:** Public health genomics, Clinical genetic testing, Public health workforce, Genomic literacy, Workforce readiness

## Abstract

**Background:**

As genetic testing becomes increasingly relevant to precision public health, understanding the preparedness of the public health workforce is essential. In the United Arab Emirates (UAE), national initiatives such as the Emirati Genome Program are progressing rapidly; however, the integration of genetic testing into public health training and practice remains limited.

**Objective:**

To examine the knowledge, attitudes, and training on genetic testing among public health professionals and students in the UAE, and to identify perceived barriers and enablers to its integration into public health practice.

**Methods:**

A qualitative descriptive design with embedded quantitative components was used. Semi‑structured interviews were conducted with 19 purposively selected public health stakeholders, including faculty members, postgraduate students, and public health professionals. Participants completed a brief pre‑interview survey capturing demographics, education, and self‑reported and objective knowledge on genetic testing. Qualitative data were analyzed using structured coding procedures, and quantitative data were summarized descriptively.

**Results:**

Training on genetic testing was limited across undergraduate and postgraduate education, and most participants rated their knowledge as insufficient. Participants demonstrated moderate understanding of analytic validity, clinical validity, clinical utility, and the existence of international guidelines. However, some knowledge questions showed noticeably lower correct‑response rates. Despite these gaps, participants expressed positive attitudes toward the relevance of genetic testing for public health. Four themes emerged: recognition of genetic testing’s value for disease prevention; sociocultural and ethical considerations influencing public acceptance; the need for enhanced education and institutional capacity; and cautious support for regulated consumer‑initiated genetic testing.

**Conclusion:**

Public health professionals and students who participated in the current study in the UAE show strong interest in genetic testing but report suboptimal training. Addressing these gaps will require systematic integration of genomics into public health curricula, targeted workforce development, and supportive policy frameworks aligned with national precision health initiatives.

**Supplementary Information:**

The online version contains supplementary material available at 10.1186/s12909-026-09262-z.

## Introduction

Chronic diseases, congenital disorders, and inherited conditions continue to impose a substantial burden on health systems worldwide, contributing to high morbidity, mortality, and escalating economic costs [1]. As genomic technologies advance, indication‑based clinical genetic testing, which evaluates individuals with a personal or family history suggestive of a hereditary condition, has become increasingly relevant to public health practice. Within the broader field of Public Health Genomics (PHG), genetic testing supports earlier detection, targeted prevention, and more tailored health interventions [2–4]. For example, clinical genetic testing for hereditary breast and ovarian cancer syndrome associated with *BRCA1* and *BRCA2* variants has enabled the identification of individuals at elevated cancer risk and informed evidence‑based prevention strategies [5, 6].

PHG aims to translate genomic discoveries into population‑oriented, equitable, and ethically grounded public health practice [7, 8]. Established applications include newborn screening for metabolic disorders, carrier screening programs, often delivered premaritally in the Eastern Mediterranean Region, for hemoglobinopathies, and pharmacogenomic testing to optimize medication response [9–11]. Large‑scale carrier screening initiatives have significantly reduced the incidence of sickle cell disease and β‑thalassemia in several countries, demonstrating the potential of genomics‑informed prevention [12]. These developments reflect a global shift toward precision public health, in which genomic, environmental, and social determinants are integrated to guide more effective and context‑specific interventions [13–18].

Despite these scientific advances, genomic literacy among public health professionals remains limited. Studies across North America, Europe, Asia, and the Middle East consistently report gaps in foundational genomic knowledge, low confidence in interpreting genetic information, and limited exposure to genomics within public health training programs [19–24]. In the United States, for example, fewer than 15% of accredited Master of Public Health programs require coursework in genomics [19]. Similar gaps have been identified in Europe and the Asia‑Pacific region, where public health practitioners describe limited training opportunities and uncertainty about how genomics aligns with their professional roles [23, 25]. Structural barriers are also reported in the Middle East and North Africa region, where genomics education remains inconsistently embedded in health professional training programs, and continuing professional development opportunities are limited [26, 27].

Successful integration of clinical genetic testing into public health practice depends not only on technological capacity but also on the preparedness of the workforce. Workforce readiness encompasses knowledge, competencies, attitudes, ethical awareness, and the ability to translate genomic information into prevention and policy strategies. Although public health professionals often express positive attitudes toward genomics, many report uncertainty about its practical application, ethical implications, and relevance to their roles [28, 29].

The United Arab Emirates (UAE) has made substantial investments in genomic infrastructure through the Emirati Genome Program, a national initiative aimed at sequencing Emirati citizens to support personalized medicine and enhance public health surveillance. However, despite this progress, the integration of clinical genetic testing into routine public health practice remains limited. Insurance coverage varies, implementation frameworks are still emerging, and workforce training pathways have not yet been standardized [30]. Experiences from other countries demonstrate that insufficient workforce capacity can hinder the effective implementation of PHG initiatives [31, 32].

Given the UAE’s strong health infrastructure and commitment to innovation, the country is well-positioned to lead regional efforts in public health genomics. Realizing this potential requires a clear understanding of the current readiness of the public health workforce. This study, therefore, aims to examine the knowledge, attitudes, education, perceived readiness, and training needs of public health professionals in the UAE regarding indication‑based clinical genetic testing, and to identify key barriers and enablers to its integration into public health practice.

## Materials and methods

### Study design

This study employed a qualitative descriptive design with an embedded quantitative component to explore public health professionals’ knowledge, attitudes, and perceived readiness regarding indication‑based clinical genetic testing. Data collection consisted of semi‑structured interviews and a brief pre‑interview online questionnaire adapted, with permission, from a previously published study conducted in Italy [31]. Although the original instrument was developed in 2014, it was selected to allow readers to compare results of the questionnaire across studies (Supplementary file 1).

### Participants

A purposive, quota-based sampling strategy was initially employed to recruit participants from five predefined stakeholder groups: (1) public health faculty members, (2) senior Master’s and PhD students in public health, (3) alumni of public health programs, and (4) government public health specialists and officers. To enhance sample diversity and ensure adequate representation of relevant expertise, snowball sampling was subsequently used. Participants were invited to recommend additional individuals who were either members of these predefined stakeholder groups or public health professionals closely aligned with the study focus through their academic, professional, or policy-related roles. All participants recruited through snowball sampling met the original eligibility criteria and were conceptually aligned with the predefined stakeholder categories. All participants were based in the UAE, and the study was conducted between January and April 2024.

### Data collection

All participants received a detailed information sheet and provided electronic informed consent before participating. The knowledge questions were adapted from the original Italian study [31] and pilot-tested for content relevance in the UAE context. However, this instrument is not formally psychometrically validated. A self-administered online questionnaire was shared at least 24 h in advance of the interviews. This questionnaire captured demographic data (gender, nationality, profession, years of experience), self-assessed knowledge of genetic testing (via a 7-item instrument), and attitudes toward its use in public health (Supplementary file 2).

Interviews were conducted remotely via secure video conferencing by a trained researcher (ATR) and followed a semi-structured guide focused on four domains: (1) knowledge and educational exposure to genetic testing, (2) perceived readiness for implementation, (3) training needs, and (4) systemic barriers and enablers. Interviews lasted between 30 and 55 min and were audio-recorded with permission. Field notes were documented during and after interviews to capture contextual cues, non-verbal observations, and interviewer reflections.

### Transcription and data validation

Audio recordings were transcribed verbatim by three team members (IEK, MA, AA). Two researchers (ATR, BH) independently reviewed the transcripts to ensure accuracy. Member checking was conducted by inviting randomly selected participants to review their transcripts and provide clarifications or corrections. This process enhanced credibility by ensuring fidelity of interpretation and also supported reflexivity, as it allowed researchers to reassess assumptions and reflect on their influence on data interpretation.

### Data analysis

Qualitative data were analyzed using the structured coding procedures described by Strauss and Corbin, including open, axial, and selective coding, consistent with a qualitative descriptive analytic approach [33]. The analysis followed iterative open, axial, and selective coding, facilitated using NVivo software. Codes were developed inductively, and categories were refined through constant comparison until final themes were established. Two researchers (ATR, BH) led the coding process and reached consensus on final themes and interpretations. Quantitative data from the pre-interview questionnaire were analyzed descriptively (frequencies, percentages), and the visualization of the results was conducted using R software.

## Results

### Participant characteristics

Nineteen individuals participated in the study, representing a diverse cross‑section of the public health workforce in the United Arab Emirates. Participants included faculty members and graduate students, researchers, public health specialists, and government-affiliated practitioners whose roles involve health promotion, surveillance, policy development, or public health program implementation, and most were expatriates residing in the UAE. Table 1 summarizes participant characteristics.

**Table 1:** Participant’s information

Gender	Professional Position in Public Health	Year of Highest Qualification	Highest Qualification	Years of Experience in Public Health
Male	Faculty Member in Public Health	2010	Ph.D	8
Male	Public Health Officer	2022	Ph.D	11
Female	Public Health Master Student	2021	MBBS	3
Female	Faculty of Member Public Health	2022	Ph.D	1
Male	Faculty Member in Public Health	2011	Ph.D	12
Female	Public Health PhD Student	2015	Master	15
Female	Public Health Researcher	2023	Ph.D	4
Male	Faculty Member in Public Health	2006	Ph.D	4
Female	Public Health PhD Student	2007	Master	7
Female	Faculty Member in Public Health	2006	Ph.D	7
Female	Faculty Member in Public Health	2017	Master	1
Female	Faculty Member in Public Health	2020	Ph.D	1
Female	Public Health Specialist	2019	Master	2
Male	Public Health PhD Student	2012	Master	17
Male	Public Health PhD Student	2018	Master	3
Male	Faculty Member in Public Health	2002	Ph.D	20
Female	Faculty Member in Public Health	2020	Ph.D	1
Female	Public Health PhD Student	2018	Master	6
Female	Public Health Specialist	2012	Master	12

### Findings from the pre-interview survey

#### Education on genetic testing

**Fig. 1:**
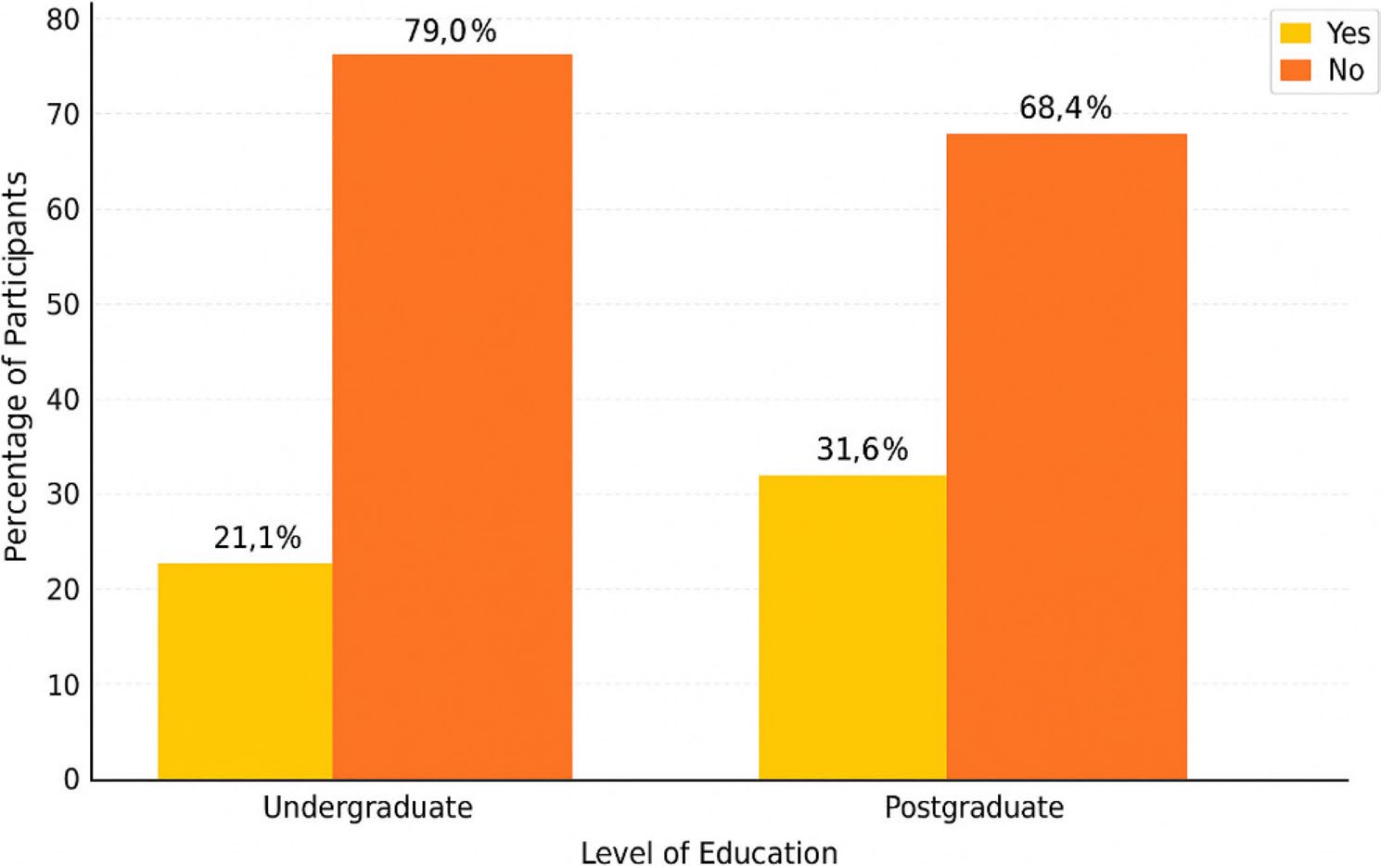
The percentage of participants reported training on genetic testing during undergraduate and postgraduate education

Formal training in clinical genetic testing was limited, and only four participants (21.1%) reported training on genetic testing during undergraduate, and six (31.6%) during postgraduate training (Fig. 1). Engagement in continuing professional education varied; eight of the participants reported 1–5 h per week, six reported less than 1 h, three reported more than 10 h, and one reported 6–10 h weekly.

#### Knowledge of genetic testing

**Fig. 2:**
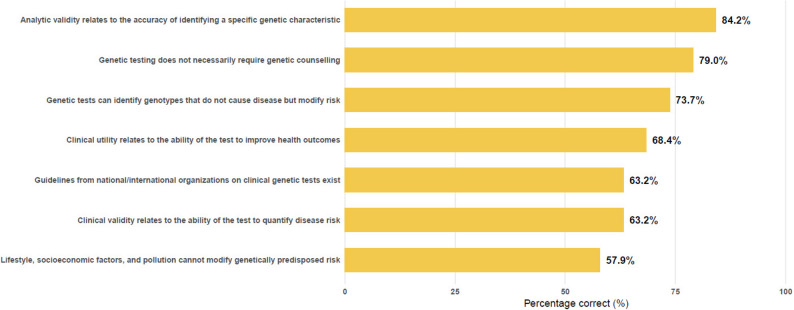
Percentage of correct responses to knowledge questions

Participants demonstrated moderate understanding of analytic validity, clinical validity, clinical utility, and the existence of international guidelines. However, some knowledge questions showed noticeably lower correct‑response rates, indicating gaps in participants’ understanding of key genomic concepts (Fig. 2). Self‑perceived knowledge was generally low. None of the participants rated their knowledge as excellent; three rated it as good, five as sufficient, and the majority (*n* = 11) reported insufficient knowledge.

#### Support for genomics education

There was a strong consensus regarding the need for formal genomics education in public health. Nearly all participants (*n* = 18) supported integrating clinical genetic testing content into undergraduate public health curricula, and all participants endorsed developing dedicated postgraduate training programs focused on genomics‑informed chronic disease prevention.

### Qualitative findings

Analysis of interview data generated four major themes reflecting participants’ perspectives on the value, challenges, and implementation considerations of genetic testing in the UAE. Table 2 provides illustrative quotes.

**Table 2:** Summary of themes and illustrative quotes

Theme	Description	Illustrative Quotes
1. Recognition of the relevance of clinical genetic testing to public health practice	Participants generally viewed genetic testing as a promising tool in public health. They expressed enthusiasm about its potential for early detection and disease prevention. However, opinions varied on which diseases should be prioritized.	*“I think yes*,* of course it will help identify future probability of disease. It is cost-effective.” (P5*,* Male*,* Faculty Member) “There is a huge prevalence of diabetes in the UAE*,* but I would start with colorectal cancer—it’s on the rise.” (P9*,* Female*,* PhD Student)*
2. Sociocultural and ethical considerations shaping acceptance	Participants acknowledged that religious beliefs and cultural norms could influence public acceptance of genetic testing, though many believed in the roles of social media and community leaders in educational efforts.	*“There are so many contradictory viewpoints—some religious*,* some cultural. This is not unique to UAE.” (P14*,* Male*,* PhD Student) “People are highly educated now. Most have Master’s or even PhDs. They will understand the value of genetic testing.” (P11*,* Female*,* Faculty Member) “Social media is not working well for genetic testing. Religious figures and influencers may help more.” (P5*,* Male*,* Faculty member)*
3. The need for enhanced education and institutional capacity	Participants emphasized the need for better integration of genetic testing in public health curricula. They also noted gaps in the public health curriculum.	*“There is not enough exposure in our curriculum… we need to increase efforts.” (P3*,* Female*,* MPH Student)*
4. Views on regulation and consumer‑initiated genetic testing	Participants expressed cautious openness to consumer‑initiated genetic tests but emphasized the need for regulatory oversight to prevent misuse.	*“Maybe allow it with a prescription—a hybrid approach where a practitioner can provide guidance.” (P13*,* Female*,* Public Health Specialist) “Allow it with restrictions. It should not be banned*,* but not freely open either.” (P12*,* Female*,* Faculty Member)*

#### Theme 1: Recognition of the relevance of clinical genetic testing to public health practice

Participants widely acknowledged the value of clinical genetic testing for early detection, risk stratification, and targeted prevention. Many emphasized its potential relevance to high‑burden conditions in the UAE. As one participant stated, “I think yes, of course it will help identify the future probability of disease. It is cost‑effective.” Despite this enthusiasm, participants differed in their views on which conditions should be prioritized for implementation. This theme reflects strong conceptual readiness and openness to genomics, suggesting that barriers to implementation are not rooted in resistance but in gaps in training and institutional support.

#### Theme 2: Sociocultural and ethical considerations shaping acceptance

Participants highlighted the influence of cultural norms, religious beliefs, and concerns about stigma on public acceptance of genetic testing. However, these factors were not viewed as insurmountable barriers. Many participants believed that rising education levels and prior exposure to screening programs could mitigate resistance. One participant noted, “People are highly educated now… They will understand the value of genetic testing.” This theme underscores the need for culturally sensitive communication strategies and ethical awareness within the public health workforce.

#### Theme 3: The need for enhanced education and institutional capacity

Participants consistently described limited exposure to genomics in their academic training and a lack of structured continuing professional development opportunities. This gap contributed to low confidence in applying genomic concepts in practice. As one participant stated, “There is not enough exposure in our curriculum… we need to increase efforts.” This theme highlights the need for systematic integration of genomics into public health education and institutional capacity‑building to support implementation.

#### Theme 4: Views on regulation and consumer‑initiated genetic testing

Participants expressed cautious support for expanding access to genetic testing but emphasized the need for regulatory oversight and professional guidance. Concerns included misinterpretation of results, psychological harm, and the absence of standardized frameworks. Many favored a hybrid model in which individuals could initiate testing, but results would be mediated by qualified professionals. As one participant suggested, “Maybe allow it with a prescription—a hybrid approach where a practitioner can provide guidance.” This theme reflects awareness of the governance and policy dimensions of genomic integration and the need for clear national guidelines.

## Discussion

This study provides one of the first empirical examinations of public health professionals’ readiness to engage with indication‑based clinical genetic testing and public health genomics in the UAE. Participants in this study demonstrated conceptual awareness of key genetic principles, such as analytic validity, clinical utility, and the role of genetic testing in disease risk identification. However, most reported insufficient knowledge and limited curricular exposure before and during their professional public health practice. These findings mirror international evidence from Europe, Asia, and North America, where public health and healthcare professionals consistently express interest in genomics but lack the training required for effective implementation [22, 31, 34].

A key finding of this study was the limited focus on genomics across undergraduate and postgraduate public health education. This gap has been previously documented among medical and health science students in the UAE, who identified a lack of training and guidance as major barriers to genomic medicine adoption [29]. Our results extend this concern to public health professionals, indicating that the educational gap persists across the continuum of training. Encouragingly, participants strongly supported integrating genomics into public health curricula, aligning with global recommendations from the WHO and regional genomics task forces calling for modernization of public health education to meet the demands of precision health [35–40].

Despite limited knowledge, participants expressed highly positive attitudes toward the integration of clinical genetic testing into public health practice, particularly for early detection and chronic disease prevention. This attitudinal readiness is consistent with findings from other countries, where public health workers recognize the potential value of genomics [31, 41]. However, as implementation research emphasizes, positive attitudes alone are insufficient. Effective integration requires coordinated institutional change, workforce development, regulatory clarity, and supportive infrastructure [42, 43]. International experience demonstrates that embedding genomics into public health practice requires tailored competency models and continuing professional development programs. The limited presence of genomics in public health programs reflects structural gaps rather than a lack of professional interest. Implementation science frameworks may guide the development of multi-level strategies addressing training, workflows, leadership, and incentives [44].

Participants also highlighted sociocultural and ethical considerations that may influence public acceptance of genetic testing, including beliefs about destiny, stigma, and concerns about misuse of genetic information. However, many noted that societal attitudes are evolving due to increased education, digital connectivity, and prior exposure to carrier screening programs. These insights align with literature documenting the importance of culturally tailored communication strategies in public health genomics. Participants emphasized the value of engaging trusted community figures, such as religious leaders and influencers, to support genomic literacy efforts. This approach is consistent with successful UAE public health campaigns, including the “War on Diabetes” initiative and COVID‑19 communication strategies [45].

Finally, participants expressed cautious support for expanding access to genetic testing but emphasized the need for regulatory oversight. Many distinguished between unregulated direct‑to‑consumer genotyping and consumer‑initiated clinical testing, in which individuals can request testing, but results are mediated by qualified professionals. This distinction aligns with global expert recommendations advocating proportional regulations that balance access, test quality, and ethical safeguards [46, 47].

### Limitations

Several limitations should be considered when interpreting the findings. The qualitative descriptive design and small, purposively selected sample limit the generalizability of the findings beyond the study context. Although efforts were made to recruit participants from varied professional backgrounds, the sample may not fully represent all public health professionals in the UAE, particularly those working outside academic or governmental settings. Self‑reported assessments of knowledge and participants’ responses may be subject to social desirability bias, and the tool was not psychometrically validated. Additionally, some items in the interview guide were inherently leading in nature.

## Conclusion

Public health professionals and students who participated in the current study in the UAE show strong interest in genetic testing but report suboptimal training. Addressing these gaps will require systematic integration of genomics into public health curricula, targeted workforce development, and supportive policy frameworks aligned with national precision health initiatives.

## Supplementary Information

Supplementary Material 1.

Supplementary Material 2.

## Data Availability

The data that support the findings of this study are available from the corresponding author, upon reasonable request.
