# Supplementary material for: Knowledge, attitudes, and training on genetic testing among public health professionals and students in the United Arab Emirates: a qualitative study
Source: BMC Med Educ. 2026 Apr 29;26:981. doi: 10.1186/s12909-026-09262-z (PMC13273977; doi:10.1186/s12909-026-09262-z)
Supplement: Supplementary file 1 — Supplementary Material 1. [file 12909_2026_9262_MOESM1_ESM.pdf]

Appendix: Semi-Structured Interview Guide

Study Title: Readiness for public health genomics in the United Arab Emirates: A qualitative assessment of public health professionals' knowledge and training needs

Purpose:

To explore the perspectives, perceived readiness, and implementation barriers related to predictive genetic testing among public health professionals in the UAE.

Section 1: Introduction and Consent

Thank you for agreeing to participate in this interview. The purpose of this study is to explore your views on predictive genetic testing and its role in public health practice in the UAE. Your insights will help inform educational, policy, and programmatic initiatives aimed at integrating genomics into health promotion and disease prevention.

The interview will take approximately 30–45 minutes. Your responses will remain confidential and used solely for research purposes. You may skip any question or stop the interview at any time.

Section 2: Main Interview Questions

A. Perceptions and Awareness

1. How do you perceive the role of predictive genetic tests in increasing prevention opportunities for chronic diseases in the UAE?
2. Can you share any specific examples or scenarios where predictive genetic tests might significantly impact disease prevention?
3. What is your general stance on the introduction and integration of predictive genetic tests into the UAE healthcare system?

B. Risk–Benefit Evaluation

4. How would you balance the potential benefits of early identification of genetic risk with uncertainties about available or effective interventions?

C. Economic and Policy Considerations

5. How important do you believe economic considerations are in determining whether predictive genetic tests should be introduced or scaled up in healthcare practices?
6. Can you discuss any concerns about relying primarily on cost-effectiveness in decision-making for genetic testing implementation?

D. Guidelines and Scientific Standards

7. In your opinion, why are evidence-based guidelines important for the appropriate use of predictive genetic tests?
8. How do you envision the process of developing and updating such guidelines to remain current with evolving scientific knowledge and ethical norms?

E. Integration into Public Health Practice

9. How do you view the integration of predictive genetic tests into broader health promotion and disease prevention strategies in the UAE?
10. Can you provide examples of how such testing might complement or enhance existing health interventions (e.g., screenings, risk communication)?

F. Ethical, Legal, and Social Considerations

11. In your opinion, how should decision-makers balance medical necessity with ethical and social implications when implementing genetic testing programs?
12. How should legal concerns be addressed, particularly issues related to privacy, discrimination, or consent?
13. Specifically, how do you view the ethical and legal implications of consent when implementing biobank initiatives in the UAE?

Section 3: Closing

14. Is there anything else you would like to add or emphasize regarding your views on predictive genetic testing or its role in UAE healthcare?

Thank you very much for your time and valuable insights.
