# Supplementary material for: Knowledge, attitudes, and training on genetic testing among public health professionals and students in the United Arab Emirates: a qualitative study
Source: BMC Med Educ. 2026 Apr 29;26:981. doi: 10.1186/s12909-026-09262-z (PMC13273977; doi:10.1186/s12909-026-09262-z)
Supplement: Supplementary file 2 — Supplementary Material 2. [file 12909_2026_9262_MOESM2_ESM.pdf]

The Demographic survey

A. PERSONAL DETAILS

A1. Birth year _________________ gender M ❒ F ❒

B. PROFESSIONAL ACTIVITY

b1. YEAR OF DEGREE ____

b2. TYPE OF DEGREE____

B3. POSTGRADUATE TRAINING (MULTIPLE ANSWERS ARE ALLOWED):
a) Postgraduate School ❒Type:____
b) Ph. D. ❒ Type:____
c) Master ❒ Type: ____
d) Advanced postgraduate ❒ Type:
____

e) Other training courses ❒ Please, specify:
____

B4. CURRENT SETTING OF PROFESSIONAL ACTIVITY
Please, specify
____
_

____
____

b5. HOW MANY YEARS do you HELD YOUR CURRENT professional activity?___________________

b6. in which CITY DO you conduct your professional activity?_____________________________

B7. HAVE PREDICTIVE GENETIC TESTING BEEN TREATED DURING YOUR UNDERGRADUATE TRAINING?

NO ❒ YES ❒

B8. HAVE PREDICTIVE GENETIC TESTING BEEN TREATED DURING YOUR POSTGRADUATE TRAINING?

NO ❒ YES ❒

C. ACCESS TO CONTINUING MEDICAL EDUCATION

C3. IS THERE A SCIENTIFIC LIBRARY AVAILABLE IN YOUR WORKPLACE?

NO ❒ YES ❒

C4. HOW MANY HOURS PER WEEK DO YOU SPEND IN CONTINUING MEDICAL EDUCATION?

<1 hour per week ❒ 1-5 hours per week ❒ 6-10 hours per week ❒

>10 hours per week ❒

C5. DURING THE LAST YEAR DID YOU RECEIVED INFORMATION MATERIAL ON PREDICTIVE GENETIC TESTING BY ANY AUTHORITIES?

C6. DURING THE LAST YEAR DID YOU RECEIVED ADVERTISING MATERIAL ON PREDICTIVE GENETIC TESTING?

NO ❒ YES ❒

IF YES, FROM WHICH SOURCES? ________________________________
